# Detection of Cyclic Diguanylate G-Octaplex Assembly and Interaction with Proteins

**DOI:** 10.1371/journal.pone.0053689

**Published:** 2013-01-07

**Authors:** Ori J. Lieberman, Jeffery J. DeStefano, Vincent T. Lee

**Affiliations:** 1 Department of Cell Biology and Molecular Genetics, University of Maryland at College Park, College Park, Maryland, United States of America; 2 Maryland Pathogen Research Institute, College Park, Maryland, United States of America; University of Texas-Huston Medical School, United States of America

## Abstract

Bacterial signaling networks control a wide variety of cellular processes including growth, metabolism, and pathogenesis. Bis-(3′–5′)-cyclic dimeric guanosine monophosphate (cdiGMP) is a secondary signaling nucleotide that controls cellulose synthesis, biofilm formation, motility and virulence in a wide range of Gram-negative bacterial species. CdiGMP is a dynamic molecule that forms different tertiary structures *in vitro*, including a trans-monomer, *cis*-monomer, *cis*-dimer and G-octaplex (G8). Although the monomer and dimer have been shown to be physiologically relevant in modulating protein activity and transcription, the biological effects of the cdiGMP G8 has not yet been described. Here, we have developed a TLC-based assay to detect radiolabeled cdiGMP G8 formation. Utilizing the radiolabeled cdiGMP G8, we have also shown a novel inhibitory interaction between the cdiGMP G8 and HIV-1 reverse transcriptase and that the cdiGMP G8 does not interact with proteins from *Pseudomonas aeruginosa* known to bind monomeric and dimeric cdiGMP. These results suggest that the radiolabeled cdiGMP G8 can be used to measure interactions between the cdiGMP G8 and cellular proteins, providing an avenue through which the biological significance of this molecule could be investigated.

## Introduction

Bis-(3′–5′)-cyclic dimeric guanosine monophosphate (cdiGMP) has become a key player in bacterial signaling networks since the first report describing its allosteric regulation of cellulose synthesis [1], [2]. CdiGMP has now been implicated in several important aspects of microbial behavior and pathogenesis including inhibition of motility, activation of biofilm formation and regulation of virulence [3], [4], [5], [6], [7].

**Figure 1:**
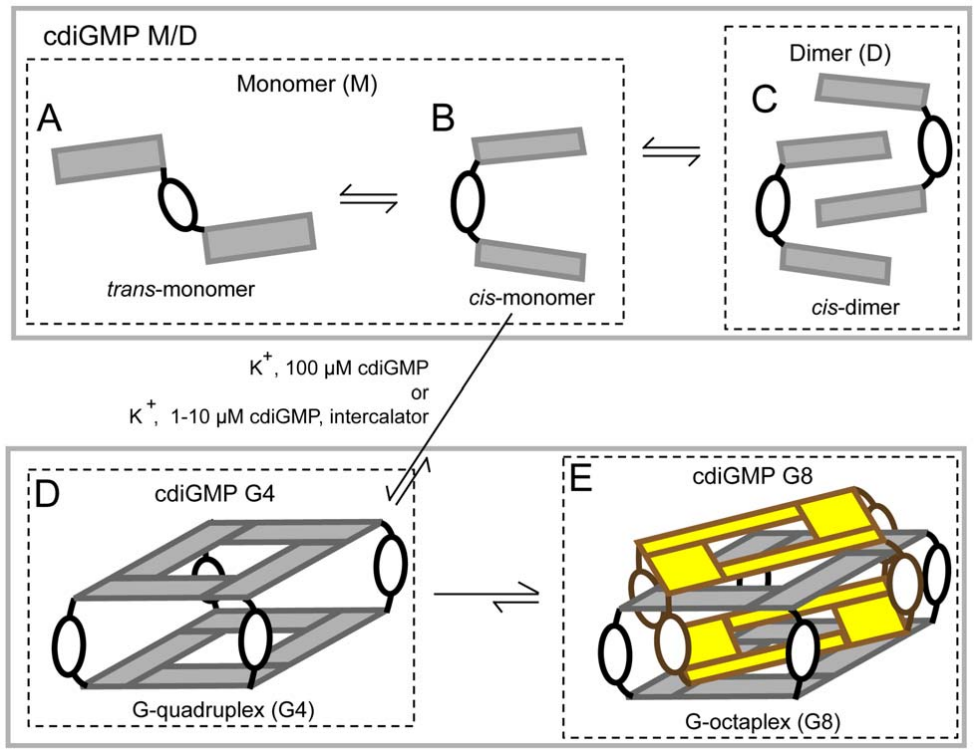
Cartoon depicting cdiGMP structures in solution. (**A**) The *trans*-monomer has the two bases oriented away from each other. (**B**) The *cis*-monomer has the two bases overlapping and is a precursor for higher order cdiGMP polymorphs. (**C**) The *cis*-dimer forms when two *cis*-monomers intercalate. This structure is stabilized by both π-stacking and hydrogen bonds between phosphates and bases. (**D**) The G-quadruplex (G4) forms when the bases from four *cis*-monomers interact via Hoogsteen base pairing and π-stacking. Monovalent cations, such as potassium, and high concentrations of cdiGMP promote this structure. The cation is located either in the middle of the G-tetrad plane or between G-tetrads and has been excluded here for clarity. (**E**) The G-octaplex (G8) forms when two G4 structures sandwich each other. This structure is stabilized by hydrogen bonding and base stacking. When potassium and high concentrations are present, the G8 is the predominant higher order rotaform.

CdiGMP is a unique signaling dinucleotide because its two bases are rotatable with respect to each other allowing the molecule to take on a number of rotational conformations (rotaforms). When made in the cell, cdiGMP affects cellular processes through direct binding to receptor proteins and RNAs [5], [8], [9]. Structural characterizations of cdiGMP interaction with macromolecules have shown to be specific for each rotaform. In the *trans* monomer form of cdiGMP, the two bases are pointing away from each other (Fig. 1A). The *trans*-monomer form has been crystallized in complex with phosphodiesterases, including FimX, BlrP1, and YkuI which are responsible for the hydrolysis of cdiGMP to pGpG [10], [11], [12]. In the *cis* form, the two bases of the monomer are rotated 90° resulting in partially overlapping of the guanine bases (Fig. 1B). The *cis*-monomer binds both VCA0042 [13], a PilZ-domain containing protein, as well as RNA riboswitches, such as Vc2 and GEMM [14], [15], [16]. In the *cis* rotaform, cdiGMP can self-assemble to form higher order complexes [17], [18], [19]. The first complex is the *cis*-dimer in which one guanine from each monomer is sandwiched between the two guanines of the other monomer (Fig. 1C) [20]. The *cis*-dimer binds to diguanylate cyclases, such as PleD and WspR, the PilZ-domain protein PA4608 and a cdiGMP-regulated transcription factor, VpsT, in *Vibrio cholerae* [21], [22], [23], [24]. The monomer and dimer forms of cdiGMP interconvert on a millisecond time scale [25]. In this manuscript, we will refer to these interchangeable forms as cdiGMP M/D. Another complex that can form from the *cis* rotaform is a G-quadruplex (G4) in which four *cis* monomers interact through base stacking and Hoogsteen base pairing [17], [18], [19], [20] (Fig. 1D). As a result, the cdiGMP G4 complex has two stacked G-tetrad layers connected by four ribose-phosphate linkages. Finally, four additional cis-monomers can intercalate into the cdiGMP G4 complex to form a G-octaplex which we refer to as cdiGMP G8 (Fig. 1E) [19], [25]. The backbones of each quadruplex are offset from each other in this structure (Fig. 1E) [19]. UV and NMR analyses of cdiGMP in solution suggest that, when complexed, only a small fraction of cdiGMP exists as a G4 but a larger fraction is complexed as a G8 [25]. The stability of the assembled cdiGMP G8 is similar to that of other known G-quadruplexes [26]. Together these studies demonstrate that many rotaforms of cdiGMP are functionally recognized by cellular protein and RNA. However, the biological function of cdiGMP G8 has yet to be described. Developing biochemical assays to detect cdiGMP G8 interactions with protein and RNA will provide important insight into the ability of cdiGMP G8 can to function in a biological setting.

G-quadruplex structures have recently come under scrutiny as they have been discovered in chromosomal DNA of both prokaryotes and eukaryotes [27], [28], [29]. In *Neisseria gonorhoeae*, G-quadruplex structures in the promoter of *pilE* have been shown to regulate antigenic variation [30]. In mammalian cells, G-quadruplex structures are present in telomere sequences to allow maintenance of chromosomal ends [31], [32], [33] and as transcription regulators of important oncogenes [34], [35], [36], [37]. Proteins, such as TEBPalpha and TEBPbeta from *Stylonychia lemnae* [38] and Hop1 from *Saccharomyces cerevisiae* [39], promote formation of G-quadruplex structures. Other proteins, including the single stranded DNA binding protein UP1, are able to unfold these structures [40]. Through systematic evolution of ligands by exponential enrichment (SELEX) selection for protein binding nucleic acid aptamers, oligonucleotides have been described that are able to bind specific proteins with high affinity [41], [42], [43], [44], [45]. Some of these aptamers contain G-quadruplex domains, including the S4 and R1T G4 aptamers that bind human immunodeficiency virus (HIV) reverse transcriptase (RT) with dissociation constant (K_d_) in the low nanomolar range and are potent inhibitors of RT activity [46], [47]. Likewise, the TBA aptamer contains a G-quadruplex and inhibits human thrombin protease at nanomolar concentrations [48], [49]. Each of these G-quadruplex containing oligonucleotides is able to bind specific proteins and modulate their activity.

The G-quadruplex domain can be formed from a diverse range of structures [26], [50], [51]. These structures are defined by: (1) intra- or intermolecular formation; (2) parallel or anti-parallel configuration of their sugar phosphate backbones; (3) *syn* or *anti* conformation of the guanosine bases with respect to the ribose in the backbone; (4) the number of layers of tetrads that compose the structure; and (5) the metal cation that interacts with the structure [26]. The cdiGMP G8 is assembled from eight cdiGMP molecules and is considered an intermolecular G-quadruplex with four tetrad layers (Fig. 1E) [18], [19]. The guanosine bases in cdiGMP G8 are either all *anti* or all *syn* [18], [19]. The cdiGMP G8 cannot be defined by the orientation of their strands because its guanosine bases are not connected by the canonical sugar phosphate backbone as in DNA and RNA. Finally, G-quadruplexes can also be defined by the conditions that promote folding. The formation of the cdiGMP G8 is dependent on potassium cation and high cdiGMP concentration above one hundred micromolar [18], [19], [25]. *In vitro,* heating and slow cooling is also required for cdiGMP G8 formation [18]. Planar intercalators, such as flavin and thiazol orange, promote cdiGMP G8 formation at lower cdiGMP concentrations (<10 micromolar), without the need for heat, by stabilizing the intermolecular complex [52], [53]. Thus, *in vitro* conditions that promote cdiGMP G8 assembly with planar intercalators can exist in the cell, indicating a possible role for cdiGMP G8 *in vivo*.

We set out to devise biochemical tools with which we could investigate whether cdiGMP G8 was able to interact with proteins specifically and modulate their activity. Here we show the generation of a radiolabeled cdiGMP G8 and its separation from radiolabeled cdiGMP M/D by thin layer chromatography. Using this newly labeled probe, we show that cdiGMP G8 can bind HIV RT specifically, but not the thrombin protease. Furthermore, we show that cdiGMP G8 is no longer recognized by known binding proteins of monomeric and *cis*-dimeric cdiGMP. Together, these results demonstrate that the potential of cdiGMP G8 to have biological function can be investigated using these techniques.

## Materials and Methods

### Materials

HIV RT (HXB2 strain) was obtained from Worthington Biochemical Corporation (Lakewood, NJ USA). Thrombin protease was purchased from GE Healthcare. CdiGMP was purchased from Axxora. T4 polynucleotide kinase (PNK) was obtained from New England Biolabs. Deoxyribonucleotide triphosphates were from Roche Applied Sciences. Radiolabeled GTP and ATP were obtained from Perkin Elmer. Oligonucleotides were synthesized from Integrated DNA Technologies including PF1–5′-AGGAAGGCTTTAGGTCTGAGATCTCGGAAT-3′, S4–5′-CGCCTGACCCTTCAGGCGTTGGGTGGGTGGGTGGG-3′, 38 NT SELEX 5′-TAATACCCCCCCTTCGGTGCAAAGCACCGAAGGGGGGG-3′, TBA25 5′-GGTTGGTGTGGTTGG-3′. Ten (10) kDa size exclusion columns were obtained from VWR. Protran nitrocellulose transfer membrane was purchased from Whatman. PEI- coated cellulose TLC plates were obtained from EMD Chemicals. Other chemicals were purchased from Sigma-Aldrich Co., Thermo Fisher Scientific, Inc., or VWR Scientific, Inc.

### Protein Purification

*P. aeruginosa* cdiGMP M/D binding protein (maltose binding protein (MBP), MBP-Alg44, MBP-PelD ΔTM, WspR D70E, RocR, MBP-PA0012, MBP-PA3353, MBP-PA4324, and MBP-PilZ) were cloned, overexpressed and purified as described in [54]. Briefly, open reading frames were cloned and overexpressed in *E. coli* BL21 (DE3). Cells were lysed and proteins were purified over a nickel-nitriloacetic acid (Ni^2+^-NTA) column. Fractions were dialyzed against 10 mM Tris, and100 mM NaCl (pH 8.0). Fractions were purified again by Q-sepharose (GE Healthcare), dialyzed again and stored in 100 mM NaCl and 10 mM Tris (pH 8.0) at −80°C until thawed for use.

### Synthesis of ^32^P-cdiGMP

^32^P-cdiGMP was synthesized by reacting 80 nM α^32^P-GTP with WspR (D70E) overnight at 37°C in 1X cdiGMP binding buffer (10 mM Tris pH 7.9, 100 mM KCl, 5 mM MgCl_2_). ^32^P-cdiGMP was separated from the WspR enzyme by passing through a 10 kDa filter and stored at −20°C [55].

### End Labeling of DNA Aptamers

Twenty-five (25) pmol of oligonucleotide (PF1), 70 mM Tris-HCl (pH = 7.6), 10 mM MgCl_2_, 5 mM dithiothreitol (DTT), 5 µL of γ-^32^P-ATP (3,000 Ci/mmol, 10 µCi/µL), and 2 µL (20 units) of T4 polynucleotide kinase were mixed in 50 µL reaction volume. The reaction was completed at 37°C for 30 minutes and PNK was then inactivated for 20 minutes at 70°C. The labeled oligonucleotide was separated from free γ-^32^P-ATP by passing over Sephadex G-25 spin column.

### CdiGMP G8 Formation

Four nM ^32^P-cdiGMP was mixed with 2 mM cdiGMP, 1X cdiGMP binding buffer (10 mM Tris base pH 7.9, 100 mM KCl, 5 mM MgCl_2_). The concentration of unlabeled cdiGMP was determined by ultraviolet absorbance. ^32^P-cdiGMP with and without unlabeled cdiGMP was then heated at 82°C for 15 minutes and allowed to cool slowly to room temperature by turning off the heat block [18], [19]. Mixtures with sodium were made in the same way except 100 mM NaCl was added to the binding buffer instead of 100 mM KCl. Unlabelled cdiGMP G8 was formed in the same way excluding addition of 4 nM ^32^P -cdiGMP. Assembled cdiGMP mixtures were stored at −20°C and thawed for experiments. Concentrations of the cdiGMP G8 referenced in this manuscript are for cdiGMP monomers that comprise each octaplex.

### Thin Layer Chromatography (TLC)

Sample (0.6 µL) was spotted on polyethylene imine (PEI) cellulose TLC plates and dried. Samples were separated with a mobile phase buffer consisting of 60% (v/v) 1.5 M KH_2_PO_4_ and 40% (v/v) saturated (NH_4_)_2_SO_4_ for 30 min [1], [56], [57], [58], [59]. The TLC plate was dried and imaged using a Fujifilm FLA-7000 phosphorimager. Images were analyzed by Multigauge software (Fujifilm). Percent conversion to G8 was calculated by dividing the intensity of the G8 band by the sum of the intensities of the cdiGMP M/D and G8 band.

### UV-vis Spectroscopy

UV-vis spectrum from 200 nm to 800 nm for each sample was determined using a Thermo Scientific NanoDrop 8000. UV-vis spectrometer was blanked with K^+^ buffer (10 mM Tris base pH 7.9, 100 mM KCl, 5 mM MgCl_2_).

### Differential Radial Capillary Action of Ligand Assay (DRaCALA)

Proteins and ligands at the indicated concentrations (^32^P-cdiGMP, ^32^P-cdiGMP G8 and ^32^P-labeled oligonucleotides) were mixed and incubated for 10 minutes at room temperature [54], [60]. For cdiGMP M/D binding protein panels, 9.8 µM WspR D70E, 4 µM MBP-PelD, 9.4 µM MBP-Alg44, 6 µM RocR, 3.9 µM MBP-PA0012, 1 µM MBP-PA3353, 3.8 µM MBP-PA4324, 13 µM MBP-PilZ, 960 nM HIV RT, and 10 µM MBP were used. For competition and IC_50_ binding assays, the indicated concentration of unlabeled competitor was added to the reaction at the same time. K_d_ was obtained by holding ^32^P-cdiGMP concentration at 500 nM and serially diluting protein concentration. Reaction mixtures (2.5 µL) were spotted on dry nitrocellulose. Membrane was dried and imaged using a Fujifilm FLA-7000 phosphorimager and analyzed with Multigauge software. Fraction bound was calculated according to [54] or as specified in the text.

### HIV RT Inhibition Assays

Assays to determine the effect of cdiGMP M/D and cdiGMP G8 complexes on RT activity were conducted essentially as described in [61]. Reactions contained substrate (1∶1.2 primer (5′- TCCCCGGGTACCGAGCTCGAATTCGCCCTATAG-3′):template (5′-TTGTAATACGACTCACTATAGGGCGAATTCGAGCTCGGTACCCGGGGATC-3′), final concentration in reactions was 50 nM in 5′-^32^P end-labeled primer). HIV-1 RT (0.25 nM), 50 mM Tris-HCl (pH = 8), 1 mM DTT, 80 mM KCl (or NaCl), 6 mM MgCl_2_, 100 µM dNTPs and 0.1 µg/µL BSA. Two types of assays were used, a time course in the presence of a fixed amount the various cdiGMP forms or assays using a fixed 5 minute time point and increasing amounts of cdiGMP forms. Values for half-maximal inhibition (IC_50_) were determined from the fixed time point assays as described in [61]. The amounts of the various cdiGMP forms used in the assays are shown in the figures and figure legends.

### WspR Activity Aassays

WspR (D70E) (1.2 µM), α-^32^P-GTP, and 500 µM of the indicated inhibitor were incubated at room temperature for the specified amount of time. Reactions were then mixed 1∶3 with 50 µM ethylenediaminetetraacetic acid (EDTA) and heated for 5 minutes at 82°C [1], [55]. Sample (0.6 µL) was then spotted on PEI-cellulose TLC plates and allowed the dry. These spots were separated with elution buffer as described above, dried and imaged as described above. Fraction cdiGMP formed was determined by measuring the intensity of the cdiGMP band and dividing by the total intensity. Measurements were made with Multigauge software.

## Results

### Higher Order Molecular Complexes of cdiGMP can be Detected by Thin-layer Chromatography

**Figure 2:**
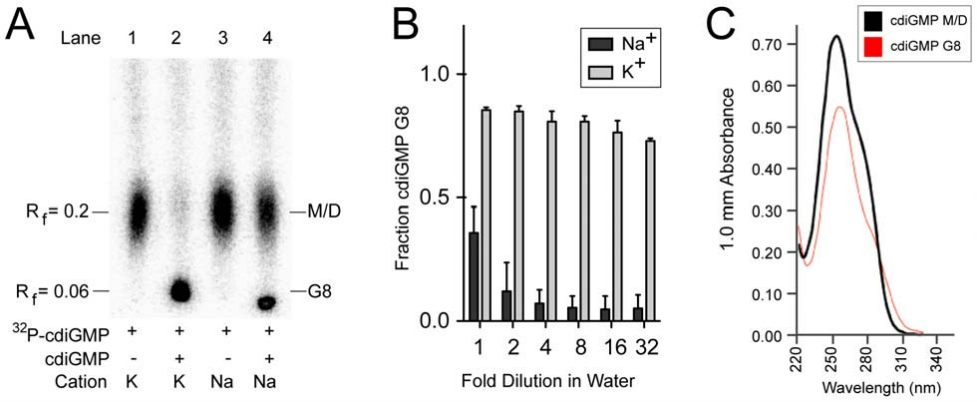
Thin layer chromatography separates ^32^P-cdiGMP G8 from ^32^P-cdiGMP M/D. (**A**) Four mixtures of ^32^P-cdiGMP were heated, cooled, spotted on PEI-cellulose TLC plates, and separated with a mobile phase of 0.9 M KH_2_PO_4_ and 40% (v/v) saturated NH_4_PO_4_. ^32^P-cdiGMP was prepared in buffer containing K^+^ (Lane 1 and 2) or Na^+^ (Lane 3 and 4) with no additional cdiGMP (Lane 1 and 3) or 500 µM cdiGMP (Lane 2 and 4). The retention factor (R_f_) is indicated on the left of the TLC. (**B**) Samples in lane 2 and 4 from Panel A were two-fold serially diluted in water and allowed to incubate at room temperature for ten minutes. Samples were then spotted and separated by TLC as in (A) and the total ^32^P-cdiGMP G8 (R_f_ = 0.06) was quantified and plotted. The first sample is undiluted and the dilution factor for the other samples is indicated on the X-axis. (C) UV-vis spectra of 500 µM cdiGMP with (red) and without (black) heat in K^+^ buffer. Absorbance was determined using a pathlength of 1.0 mm.

TLC is able to distinguish GTP, cdiGMP M/D, and pGpG [1], [56], [57], [58], [62]. We were interested in seeing whether TLC could also separate the ^32^P-cdiGMP G8 from the ^32^P-cdiGMP M/D. Generation and separation of a radiolabeled cdiGMP G8 would allow for biochemical analysis of the behavior of this molecule. To investigate whether the ^32^P-cdiGMP G8 could be separated from the ^32^P-cdiGMP M/D, four mixtures of cdiGMP were prepared under different conditions, spotted on PEI-coated TLC plates, and separated as indicated in Materials and Methods. Previous studies of cdiGMP G8 synthesis suggest that, in the absence of intercalators, G8 formation is driven by two key parameters: 1. cation type and 2. cdiGMP concentration [18], [19]. We asked whether cdiGMP G8 formation is only observed in the presence of K^+^ and high concentrations of cdiGMP when assayed by TLC. At nanomolar concentrations of cdiGMP in buffer containing K^+^, ^32^P-cdiGMP migrated with an R_f_ value of 0.2 (Fig. 2A, lane 1). In K^+^ buffer and at 500 µM cdiGMP, ∼90% of the radiolabelled cdiGMP migrated with an R_f_ of 0.06 with a minor species migrating at an R_f_ of 0.2 (Fig. 2A, lane 2). In Na^+^ buffer with nanomolar cdiGMP concentrations, ^32^P-cdiGMP migrated at an R_f_ of 0.2 (Fig. 2A, lane 3). In Na^+^ buffer with 500 µM cdiGMP, ∼40% of ^32^P-cdiGMP migrated with an R_f_ value of 0.06 while the remainder migrated to the more mobile R_f_ value of 0.2 (Fig. 2A, lane 4). The conditions that promote cdiGMP G8 formation resulted in the appearance of a slower migrating species with an R_f_ value of 0.06 when assessed by TLC [18], [19], [25].

Although we expected the ^32^P-cdiGMP to form G8 complexes in the presence of K^+^ (Fig. 1A, lane 2), a substantial portion of the ^32^P-cdiGMP in Na^+^ buffer with high cdiGMP concentration was also converted to a slower migrating species (Fig. 2A, lane 4). Since cdiGMP G8 is stable, it is resistant to disassembly into M/D forms upon dilution in water [25]. In fact, on the time scale of our experiments, we observed no interchange between the cdiGMP G8 species and M/D species when analyzed by TLC (Fig. S1). To test the G8 status of the slower migrating species observed in the Na^+^ buffer, we serially diluted both Na^+32^P-cdiGMP G8 and K^+32^P-cdiGMP G8 in water and measured the amount of ^32^P-cdiGMP G8 present by TLC after 10 minutes incubation at room temperature (Fig. 2B). Diluting the Na^+32^P-cdiGMP G8 by two-fold led to disassembly of ^32^P-cdiGMP G8 into ^32^P-cdiGMP M/D while the K^+32^P-cdiGMP G8 was stable even when diluted 32-fold (Fig. 2B). These results demonstrate that the slower migrating species observed in the K^+^ buffer is indeed assembled cdiGMP G8.

Previous analyses of cdiGMP mixtures have shown that the cdiGMP G8 has unique spectral properties in the ultraviolet spectrum with a maximum absorbance at 256 nm and a shoulder at 295 nm [18]. In contrast, the UV spectrum of cdiGMP M/D is similar to that of GTP with a maximum absorbance at 253 nm and a shoulder at 280 nm [18]. These previous studies also showed that cdiGMP G8 has a reduced maximum absorbance compared to the cdiGMP M/D [18] and the amount of cdiGMP G8 present in a sample inversely correlates to the ratio of the absorbance at 276 nm and 289 nm [25]. To determine if the cdiGMP G8 we prepared has similar spectroscopic properties, UV spectra were obtained for the sample of 500 µM cdiGMP heated and slowly cooled in K^+^ to assemble cdiGMP G8 and a sample of 500 µM cdiGMP in K^+^ that was not heated and remained cdiGMP M/D (Fig. 2C). We observed peak absorbance for the cdiGMP M/D at 253 nm while the cdiGMP G8 had a peak at 256 nm (Table 1). The maximum absorbance of the cdiGMP M/D was 0.732±0.019 while that of the cdiGMP G8 was 0.560±0.005 (Table 1). The cdiGMP M/D had an A_276_/A_289_ of 2.08±0.02 compared with an A_276_/A_289_ of 1.50±0.01 for the cdiGMP G8 (Table 1). This provides further confirmation that the cdiGMP G8 is present in samples prepared with K^+^ and runs with an R_f_ of 0.06 on TLC. Together these results show that the cdiGMP G8 can be radiolabeled and detected by TLC.

**Table 1:** Summary of UV-Vis absorbance measurements for the cdiGMP G8 and the cdiGMP M/D.

Maximum absorbance		
Sample	Wavelength (nm)	1.0 mm Absorbance
cdiGMP M/D	253	0.732±0.019
cdiGMP G8	256	0.560±0.005
**Absorbance at 276 nm**		
**Sample**	**1.0 mm Absorbance**	
cdiGMP M/D	0.453±0.012	
cdiGMP G8	0.305±0.004	
**Absorbance at 289 nm**		
**Sample**	**1.0 mm Absorbance**	
cdiGMP M/D	0.218±0.006	
cdiGMP G8	0.203±0.004	
**A_276_/A_289_**		
**Sample**	**A_276_/A_289_**	
cdiGMP M/D	2.08±0.02	
cdiGMP G8	1.50±0.01	

### Specificity of cdiGMP G8 Binding to G-quadruplex Binding Proteins

**Figure 3:**
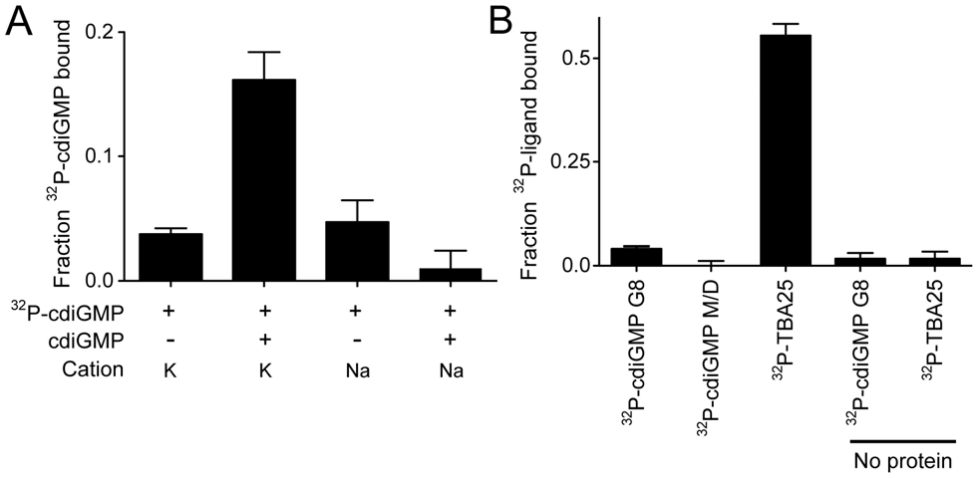
CdiGMP G8 binds HIV-1 reverse transcriptase, a parallel G-tetrad binding protein, but not thrombin protease, an anti-parallel binding protein. (**A**) HIV-1 RT (960 nM) was incubated with cdiGMP heated in 4 nM cdiGMP (−) and 500 µM cdiGMP (+) in the salt indicated. Binding was analyzed via DRaCALA. (**B**) Thrombin protease (8 µM) was incubated with ^32^P-cdiGMP G8 (lane 1), ^32^P-cdiGMP M/D (lane 2), ^32^P-TBA25 (lane 3) and binding was analyzed via DRaCALA. ^32^P-cdiGMP G8 (lane 4) and ^32^P-TBA25 (lane 5) were spotted without protein as negative controls.

Oligonucleotide aptamers identified for HIV RT and thrombin protease by SELEX revealed sequences containing G-quadruplexes. The HIV RT binds parallel G-quadruplexes while thrombin protease binds anti-parallel G-quadruplexes [41], [42], [43], [44], [45]. To test whether cdiGMP G8 can be directly bound by either class of G-quadruplex binding proteins, we employed the same four mixtures described in Fig. 2A using the differential radial capillary action of ligand assay (DRaCALA) [54]. CdiGMP G8 bound RT (Fig. 3A, lane 2). In contrast, cdiGMP M/D in K^+^ buffer did not bind HIV RT (Fig. 3A, lane 1). CdiGMP prepared in Na^+^ with either low or high cdiGMP concentration was not able to bind HIV RT (Fig. 3A, lanes 3 and 4, respectively). These results suggest that the cdiGMP G8 binds to HIV-1 RT, while the cdiGMP M/D does not. Since HIV RT and thrombin protease bind G-quadruplex aptamers with parallel and anti-parallel structures, respectively [26], we asked whether cdiGMP G8 mimics either parallel, anti-parallel, or both configurations as it lacks the canonical sugar phosphate backbone. Neither the cdiGMP G8 nor the cdiGMP M/D bound thrombin protease (Fig. 3B). As a control, thrombin did bind the specific anti-parallel G-quadruplex aptamer TBA25 (Fig. 3B) [48], [49]. These results suggest that cdiGMP G8 is able to bind proteins as a conformational mimic to the parallel G-quadruplex aptamer.

### Relative Affinity of cdiGMP G8 Binding to HIV RT

**Figure 4:**
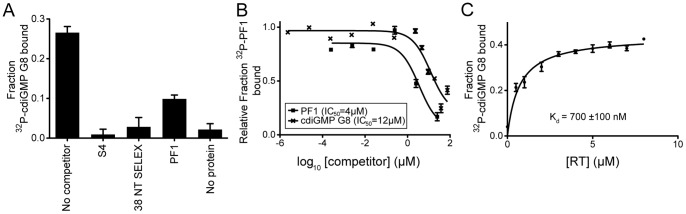
CdiGMP G8 binds to RT specifically with sub-micromolar affinity. (**A**) ^32^P-cdiGMP G8 (500 nM) was incubated with 960 nM HIV-1 RT and 5 µM indicated competitor and binding was analyzed by DRaCALA. (**B**) ^32^P-PF1 (5 nM) was incubated with 960 nM HIV-1 RT and competitor at the indicated concentrations. Binding was analyzed via DRaCALA and data was analyzed by Prism Software to obtain IC_50_ values. (**C**) ^32^P-cdiGMP G8 (500 nM) was incubated with indicated concentration of HIV-1 RT and binding was assayed via DRaCALA. The data was plotted and analyzed using Prism Software.

CdiGMP G8 could bind HIV RT in the primer-template binding site or elsewhere on the protein. To distinguish between these two possibilities, we took advantage of available aptamers S4, 38 NT SELEX, and PF1 that bind in the primer-template binding site of HIV RT with dissociation constants of approximately 1, 6 and 125 nM, respectively [47], [61], [63], [64]. By competitive binding assays using DRaCALA, addition of 10X molar excess of each unlabeled aptamer strongly reduced the interaction of RT and ^32^P-cdiGMP G8 [54], [60] (Fig. 4A). In the absence of competitor, the ^32^P-cdiGMP G8 was able to bind to RT. This suggests that the ^32^P-cdiGMP G8 binds at the primer-template binding site.

The affinity of cdiGMP G8 for HIV RT was determined using two methods. First, we determined the IC_50_ values for the inhibition of radiolabeled PF1 binding to RT by unlabeled cdiGMP G8 or PF1 aptamer. Unlabeled PF1 inhibited ^32^P-PF1 binding with an IC_50_ of 4 µM and unlabeled cdiGMP G8 inhibited with an IC_50_ of 12 µM, suggesting that the cdiGMP G8 may bind with approximately 3 times lower affinity to RT (Fig. 4B). Based on the dissociation constant (K_d_) of the PF1 aptamer for RT (∼ 125 nM) [61], the K_d_ of the cdiGMP G8 for HIV RT can be approximated as 400 nM. The K_d_ of the cdiGMP G8 and RT was also determined by direct binding using DRaCALA. By serially diluting the protein concentration and limiting ^32^P-cdiGMP G8 concentrations to 500 nM, the K_d_ can be obtained from the plot of fraction bound against the protein concentration. The K_d_ of this interaction was 700±100 nM, which is similar to the relative affinity calculated from the competitive binding assays (Fig. 4C).

### CdiGMP G8 Inhibits HIV RT Activity

**Figure 5:**
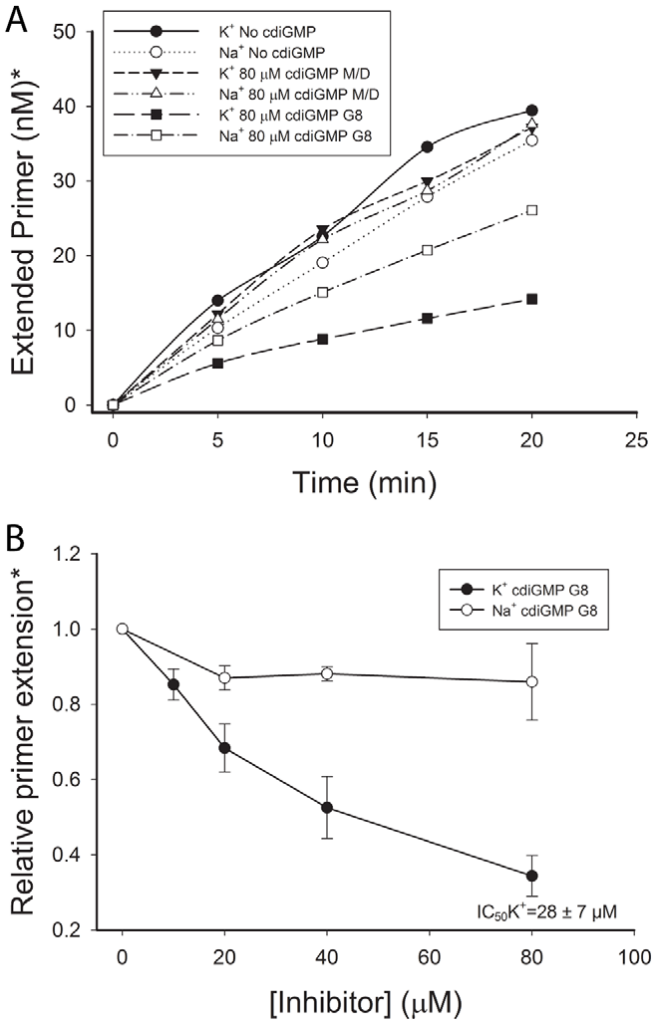
CdiGMP G8 inhibits HIV RT activity. (**A**) A graph of primer extension *vs.* time is shown for reactions conducted in the absence of presence of the indicated cdiGMP forms. Assays were performed as described in Materials and Methods. A fixed concentration of 80 µM cdiGMP form was used. Assays were conducted in either K^+^ or Na^+^ containing buffer depending on which cation was used in the preparation of the cdiGMP form. The experiment was repeated with similar results. (**B**) The amount of primer extension (5 minute time point), relative to assays conducted without added cdiGMP derivatives, *vs.* the concentration of cdiGMP G8 is shown. The graph is from an average of 3 experiments and error bars represent standard deviations. The IC_50_ value in the presence of cdiGMP G8 prepared in K^+^ was determined as described in Materials and Methods. *The concentration of extended primer in (**A**) and the relative level of primer extension in (**B**) were determined by exposure of dried denaturing acrylamide gels using a phosphoimager. Reactions contained a total of 50 nM primer.

G-quadruplex aptamers are able to bind to RT and inhibit RT activity with IC_50_ values in the nanomolar range [46], [47], [61], [63], [64]. Having demonstrated that the cdiGMP G8 binds to HIV RT at the primer-template site, we asked whether the cdiGMP G8 was able to similarly inhibit RT function. To test this, an RT primer extension assay was used [61]. Reactions included either KCl or NaCl (80 mM final concentration) depending on which cation was used in the preparation of cdiGMP G8 material. Controls with no cdiGMP or with cdiGMP M/D were also conducted. A pilot experiment showed that cdiGMP G8 prepared in K^+^ significantly inhibited RT activity when present in reactions at approximately 80 µM (data not shown). This level was chosen for the examination of various cdiGMP preparations (see Fig. 2) over a 20 minute time course assay as shown (Fig. 5A). M/D forms did not affect RT activity in reactions containing either Na^+^ or K^+^. CdiGMP G8 prepared in Na^+^ buffer showed some inhibition while material prepared in K^+^ buffer was a more potent inhibitor leading to about a 3-fold reduction in RT activity. These results suggest that the cdiGMP G8 complexes are responsible for inhibition as formation of these complexes is enhanced by K^+^, whereas the complexes formed in Na^+^ buffer disassembled into M/D upon dilution. A second set of experiments was conducted in which reactions were titrated with cdiGMP G8 complexes prepared in K^+^ or Na^+^ (Fig. 5B). This approach allowed us to determine an IC_50_ value of 28±7 µM (ave. of 3 exp. ± standard deviation) for cdiGMP G8 prepared in K^+^. A value for material prepared in Na^+^ could not be determined as the level of inhibition was too low. Although the cdiGMP G8 inhibited RT with approximately 1000X less potency than the aptamers S4, 38 NT SELEX and PF1 [47], [61], [63], [64], equimolar amounts of cdiGMP M/D showed no inhibition. These experiments establish the first report of cdiGMP G8 modulating protein function separately from cdiGMP M/D forms and suggest that the radiolabeled cdiGMP G8 can be used to biochemically investigate the physiological significance of the molecule.

### CdiGMP G8 does not Bind *Pseudomonas aeruginosa* cdiGMP M/D Binding Proteins *in vitro*

**Figure 6:**
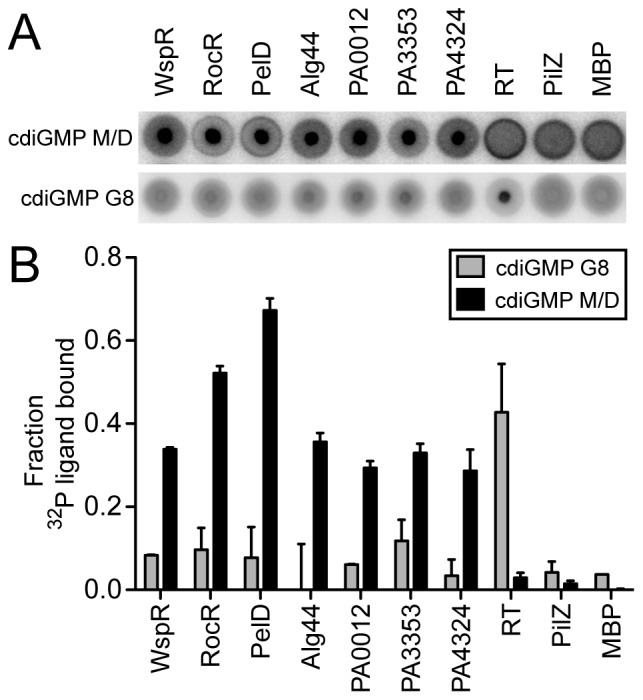
The cdiGMP G8 does not interact with known cdiGMP M/D binding proteins. (**A**) Representative DRaCALA spots for binding reactions of either ^32^P-cdiGMP M/D or ^32^P-cdiGMP G8 with the indicated protein. Binding was assayed by DRaCALA. (**B**) Quantification of fraction bound from DRaCALA of ^32^P-cdiGMP G8 reactions (gray) and ^32^P-cdiGMP M/D reactions (black).

CdiGMP is a secondary signaling molecule that regulates the behavior of many bacterial species [7], [65], [66], [67], [68]. Using the radiolabeled cdiGMP G8, we were interested in whether the cdiGMP G8 can interact with proteins known to bind cdiGMP M/D. A panel of binding proteins was tested including the WspR diguanylate cyclase that binds the *cis* cdiGMP dimer, the RocR phophodiesterase that binds *trans* monomeric cdiGMP, PelD that binds cdiGMP at a site that resembles the inhibitory site (I-site) found in many diguanylate cyclases and PilZ-domain containing proteins that bind either monomeric or dimeric cdiGMP [22], [55], [68], [69]. The ability of purified proteins to interact with either ^32^P-cdiGMP M/D or ^32^P-cdiGMP G8 were assessed by DRaCALA [54]. Interestingly, upon spotting the radiolabelled G8 mixture with no protein, two concentric circles were observed (Fig. S2). We obtained a diffuse and uniform spot on nitrocellulose upon spotting the ^32^P-cdiGMP M/D without protein (Fig. S3B). An in-depth explanation of this phenomenon and rationale behind the calculation of ^32^P-cdiGMP G8 bound can be found in the supplemental material (Fig. S2, S3, S4). Our calculations suggest that none of the bacterial cdiGMP M/D binding proteins bound ^32^P-cdiGMP G8 in contrast to the HIV RT positive control (Fig. 6A). *P. aeruginosa* cdiGMP M/D binding proteins bound ^32^P-cdiGMP M/D, whereas HIV RT did not (Fig. 6B). As a negative control for binding, maltose binding protein (MBP) and PilZ protein (PA2960), which are known not to bind ^32^P-cdiGMP M/D [69], also do not bind ^32^P-cdiGMP G8. These results indicate that cdiGMP G8 structures are no longer recognized by the same binding pockets that interact with cdiGMP M/D, and provide evidence that DRaCALA is able to measure protein binding to the cdiGMP G8.

### CdiGMP G8 is Unable to Inhibit Diguanylate Cyclase Activity

**Figure 7:**
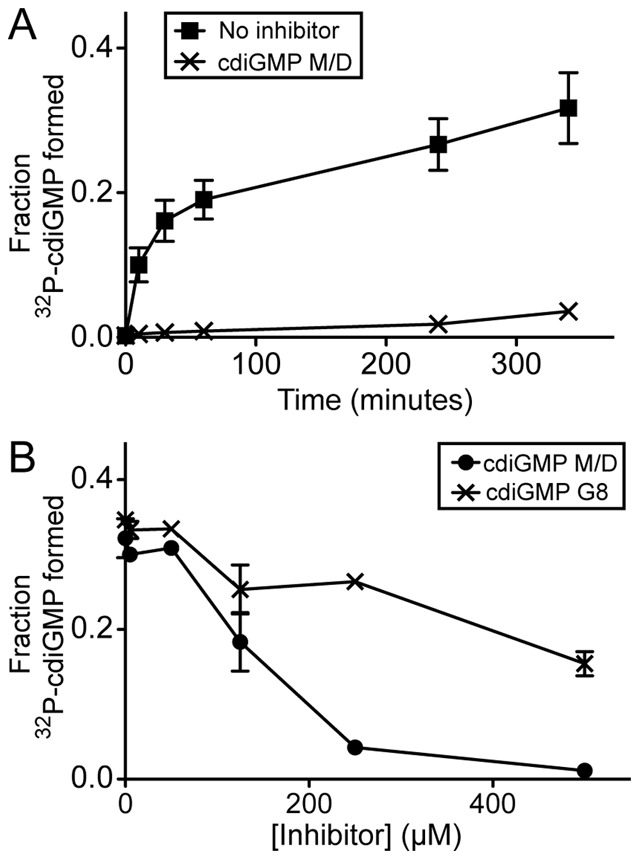
The cdiGMP G8 does not inhibit diguanylate cyclase activity in WspR D70E. (**A**) WspR D70E (1.18 µM) was incubated with 4 nM α-^32^P-GTP with and without 500 µM cdiGMP M/D. Reaction products were assayed by TLC as described in Materials and Methods and the amounts of ^32^P-cdiGMP M/D formed at specified time points were plotted. (**B**) WspR D70E (1.18 µM) was incubated with varying amounts of cdiGMP G8 or cdiGMP M/D and the fraction ^32^P-cdiGMP M/D at 3 hours was assayed by TLC and plotted.

The diguanylate cyclase WspR (PA3702) is allosterically inhibited by the cdiGMP *cis*-dimer at a regulatory I-site on the protein [22]. The I-site consists of a RxxD motif that is found in a wide range of diguanylate cyclases, which suggests that this type of inhibition is functionally conserved. An activation domain mutant version (D70E) of *wspR* has been previously described to allow for constitutive diguanylate cyclase activity [55]. We performed a time course experiment to determine the optimal time to observe cdiGMP M/D mediated inhibition of WspR D70E activity. At three hours of incubation, WspR catalyzed the maximal conversion of ^32^P-GTP to ^32^P-cdiGMP (Fig. 7A). In the presence of 500 µM cdiGMP M/D, none of the ^32^P-GTP was converted to ^32^P-cdiGMP (Fig. 7A). To investigate the ability of the cdiGMP G8 to alter WspR activity, we assayed for inhibition of varying concentrations of cdiGMP G8 or cdiGMP M/D at the three hour time point. Inhibition of WspR by cdiGMP M/D was concentration dependent with a reduction of activity by 28.3-fold at 500 µM (Fig. 7B). In contrast, the cdiGMP G8 reduced WspR activity by 2.2-fold (Fig. 7B). Residual inhibition can be attributed to remaining cdiGMP M/D in the cdiGMP G8 reaction mixture. Thus, cdiGMP G8 is unable to bind WspR or allosterically inhibit cdiGMP synthesis as observed for the cdiGMP M/D, confirming the findings of our binding studies that show that the cdiGMP G8 does not interact with the diguanylate cyclase WspR.

## Discussion

CdiGMP is a second messenger that controls biofilm formation, virulence, motility and other metabolic processes in a number of bacterial species. *In vitro*, cdiGMP can form higher order molecular structures including G8 when cdiGMP reaches concentrations greater than 100 µM, potassium is present, and the sample is heated and slowly cooled [18], [19], [25]. Although the cdiGMP G8 has been shown to form *in vitro*, no studies have confirmed its presence under physiological conditions. Planar intercalators, such as flavin rings, however, promote cdiGMP G8 formation at 1–10 µM concentrations of cdiGMP, without the requirement for heat, providing a mechanism through which the cdiGMP G8 may form in the cell [52], [53]. The biological role of cdiGMP G8 can be demonstrated in three ways: 1. visualizing cdiGMP G8 formation *in vivo*, 2. purifying cdiGMP G8 from the cell and determining its structure *in vitro*, or 3. identifying proteins that specifically interact with the cdiGMP G8 but not cdiGMP M/D. At this current time, visualization or extraction of cdiGMP G8 is not technically feasible. So, we set out to develop novel biochemical approaches to assess cdiGMP G8 interactions with proteins. These techniques could allow for investigation of the biological significance of this intermolecular complex.

Currently, the assembly of cdiGMP into G8 has been demonstrated by the appearance of a characteristic positive shift at 250 nm, a negative shift at 280 nm, an extremely strong positive shift at 215 nm and moderate positive shift at 309 nm when detected by CD spectroscopy [18]. This approach cannot simultaneously detect and quantitate both the M/D and G8 species of cdiGMP. Furthermore, binding interactions of unlabeled cdiGMP G8 with proteins would be difficult to detect by CD, due to interferences from protein signals. We utilized thin layer chromatography (TLC) to measure radiolabelled cdiGMP G8 formation *in vitro*. TLC was able to separate the ^32^P-cdiGMP G8 from ^32^P-cdiGMP M/D both qualitatively and quantitatively. Additionally, we have demonstrated that heating and cooling of cdiGMP is necessary for potassium-dependent G8 formation using this technique. This is an important development as assessing ^32^P-cdiGMP G8 function *in vitro* hinges on techniques that utilize easily detected radioactive tracers. Furthermore, validating a method that can measure the formation of cdiGMP G8 is an important step in finding interacting proteins in the cell.

How the cdiGMP G8 behaves in a biological setting will provide insight into its potential as a physiologically relevant complex. Because no cdiGMP G8 binding proteins have been described, we were first interested in finding a protein that specifically interacts with this complex. Previously, proteins such as HIV RT and thrombin protease have been shown to bind G-quadruplex structures [44], [45]. We hypothesized that these proteins may also interact with the cdiGMP G8. We have shown here that the cdiGMP G8 binds to HIV-1 RT at the primer-template binding site with K_d_ of approximately 400–700 nM. This is the first report, to our knowledge, of a cdiGMP G8 binding protein. This binding interaction can be utilized in future studies of the cdiGMP G8. G-quadruplexes can assemble either parallel or anti-parallel configurations depending on the relative arrangement of the sugar phosphate backbone [26]. We asked whether cdiGMP G8, a symmetric molecule which lacks orientation derived from the sugar-phosphate backbone, acquires properties of parallel or anti-parallel G-quadruplex structures. All previously described G-quadruplex aptamers that interact with HIV RT have been parallel-folded G-quadruplexes [61]. These RT aptamers (e.g. PF1) bind at or near the primer-template binding pocket based on their ability to compete for a common binding site with the primer-template aptamer, 38 NT SELEX [61]. The fact that the cdiGMP G8 can displace PF1 suggests it binds at the same site. Further, the fact that cdiGMP G8 binds RT suggests that it behaves as a parallel G-quadruplex. We also indirectly tested whether the cdiGMP G8 takes on an anti-parallel conformation by measuring cdiGMP G8 interaction with thrombin protease, a protein that binds an antiparallel G-quadruplex structure in the TBA aptamer. The cdiGMP G8 did not bind to thrombin. These results suggest that, despite lacking the directionality imparted by a sugar-phosphate backbone, cdiGMP G8 can take on properties of one specific configuration of G-quadruplex. These insights provide evidence that cdiGMP G8 is a unique ligand that may mediate biological processes through parallel G-quadruplex binding proteins.

Different conformations of cdiGMP structures have been shown to play a biological role in cell signaling. In *P. aeruginosa*, cdiGMP binds to a wide variety of binding pockets including the PelD, RxxD, and PilZ domain [22], [55], [69], [70]. We wondered whether the cdiGMP G8 could bind to proteins with these domains. Using the radiolabeled cdiGMP G8 that we generated, we assayed a panel of cdiGMP M/D proteins for their ability to bind the cdiGMP G8. CdiGMP G8 did not bind to any of the cdiGMP M/D binding proteins. This is the first time, to our knowledge, that the cdiGMP G8 has been shown to be a biochemically distinct ligand from the cdiGMP M/D. Future studies of the cdiGMP G8 can focus on proteins other than previously described cdiGMP M/D proteins. Finally, showing that the radiolabeled cdiGMP G8 that we have generated specifically interacts with G-quadruplex binding proteins but not cdiGMP M/D binding proteins further supports the idea that this is a viable approach in searching for cognate cdiGMP G8 binding proteins.

## Supporting Information

Figure S1 **CdiGMP G8 and cdiGMP M/D do not interchange on the experimental timescale as measured by TLC. (A)** Scanned TLC plate of 0.6 µL aliquots of cdiGMP G8 taken at time points indicated. Samples were separated as described in the Materials and Methods. **(B)** Graph of quantified fraction cdiGMP G8 and fraction cdiGMP M/D over the time scale of the experiments. Error bars indicate the standard deviation of two individual experiments. **(C)** TLC plate of aliquots from cdiGMP M/D taken from sample at indicated times. **(D)** Quantified fraction cdiGMP G8 and fraction cdiGMP M/D at indicated time points.
(TIF)

Figure S2 **^32^P-cdiGMP G8 forms two concentric circles when spotted on nitrocellulose.** **(A)** Schematic representing ^32^P-cdiGMP G8 spotting on DRaCALA. A 2.5 µL spot of radiolabel without protein is spotted and spreads out radially by capillary action leaving two circles. The ^32^P-cdiGMP G8 is present in the inner circle while the ^32^P-cdiGMP M/D is present in both. Box 4, top view shows imaged nitrocellulose after spotting. **(B)** Diagram representing diffuse radial capillary action of ^32^P-cdiGMP M/D upon spotting on nitrocellulose. One homogenous circle is observed. Box 4, top view shows an image of ^32^P-cdiGMP M/D spot (2.5 µL) on nitrocellulose. **(C)** Equation used to calculate the fraction (F) of ^32^P-cdiGMP G8 in the mixture.
(TIF)

Figure S3 **Maltose binding protein (MBP) can be used as a standard to fraction ^32^P-cdiGMP G8 bound by other proteins.** **(A)** Schematic representing appearance of DRaCALA spot upon addition of protein to the reaction mixture. Inner circle contains sequestered ligand. **(B)** Imaged DRaCALA spots (2.5 µL) of reactions that included MBP, PA3353, and RT. The line appearing through the image is used for the intensity vs. position plot in (C). **(C)** Intensity vs. position plots across the black lines in Fig. S2B for binding reactions that included MBP, PA3353, and RT. **(D)** Cartoon representing idealized intensity vs. position diagrams MBP, PA3353, and RT binding reactions.
(TIF)

Figure S4 **Equations used to calculate fraction ^32^P-cdiGMP G8 bound by cdiGMP M/D binding proteins.** **(A)** Equations used to subtract free ^32^P-cdiGMP M/D from the intensity of the entire spot. **(B)** Correction used to eliminate intensity of MBP inner circle when calculating fraction bound. **(C)** The total bound intensity is comprised of the intensity of ^32^P-cdiGMP M/D bound and the intensity of the ^32^P-cdiGMP G8 bound. **(D)** The intensity of the ^32^P-cdiGMP M/D bound can be calculated by subtracting the total unbound ^32^P-cdiGMP M/D in a sample from the total ^32^P-cdiGMP M/D in the MBP sample. **(E)** Equation used to correct for free ^32^P-cdiGMP G8 that is present in the inner circle. **(F)** Calculation used to determine the fraction ^32^P-cdiGMP G8 bound.
(TIF)

Text S1. (DOCX)
