# Correction: Detection of Cyclic Diguanylate G-Octaplex Assembly and Interaction with Proteins

**DOI:** 10.1371/annotation/1e2e5c25-79ad-4cf2-b619-f6972eafbadc

**Published:** 2013-06-07

**Authors:** Ori J. Lieberman, Jeffrey J. DeStefano, Vincent T. Lee

The second author's name was spelled incorrectly. The correct name is: Jeffrey J. DeStefano. 

