# Supplementary figures and images for: Detection of Cyclic Diguanylate G-Octaplex Assembly and Interaction with Proteins
Source: PLoS One. 2013 Jan 7;8(1):e53689. doi: 10.1371/journal.pone.0053689 (PMC3538687; doi:10.1371/journal.pone.0053689)

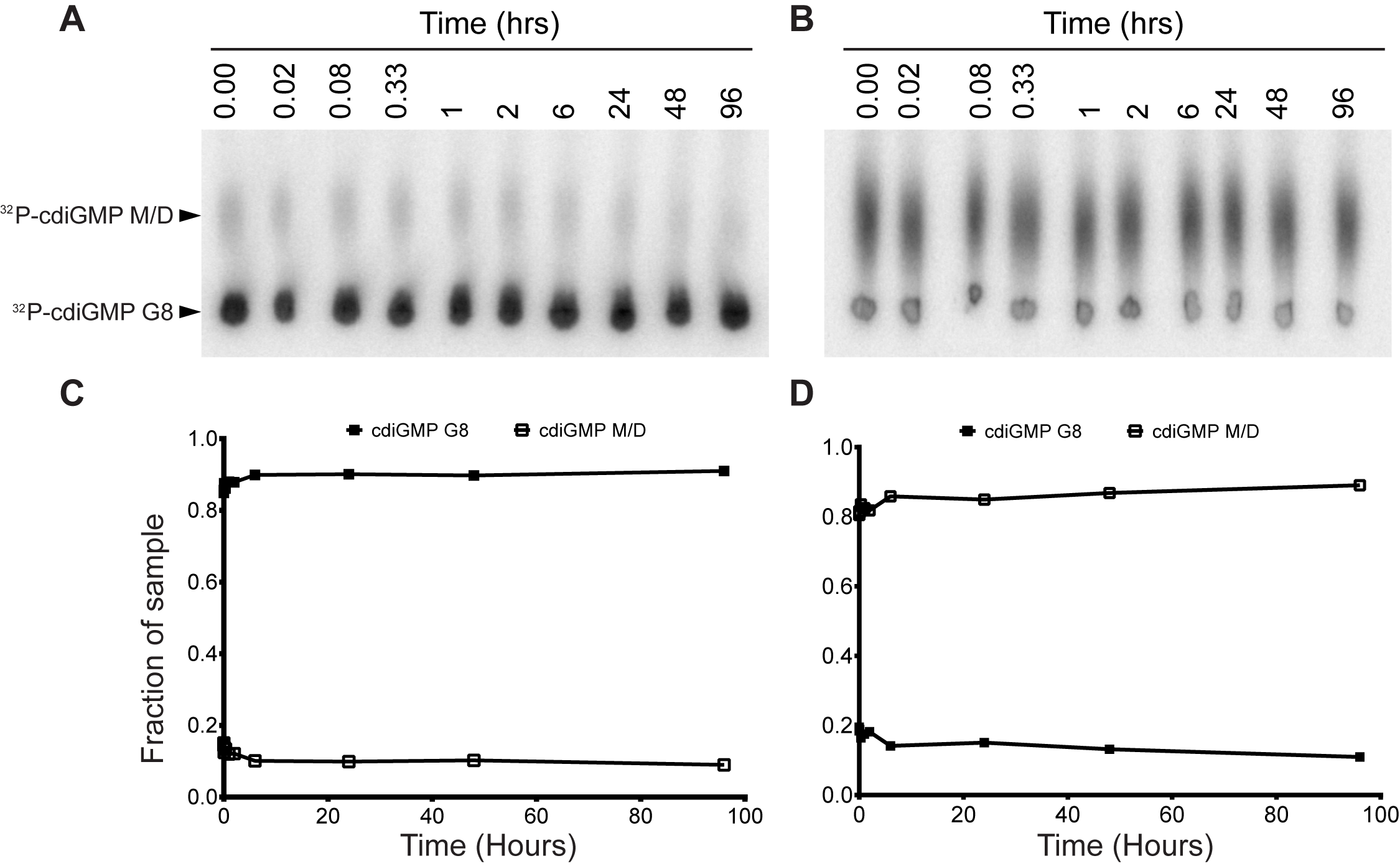

Supplement: Figure S1 — CdiGMP G8 and cdiGMP M/D do not interchange on the experimental timescale as measured by TLC. (A) Scanned TLC plate of 0.6 µL aliquots of cdiGMP G8 taken at time points indicated. Samples were separated as described in the Materials and Methods. (B) Graph of quantified fraction cdiGMP G8 and fraction cdiGMP M/D over the time scale of the experiments. Error bars indicate the standard deviation of two individual experiments. (C) TLC plate of aliquots from cdiGMP M/D taken from sample at indicated times. (D) Quantified fraction cdiGMP G8 and fraction cdiGMP M/D at indicated time points. (TIF) [file pone.0053689.s001.tif]

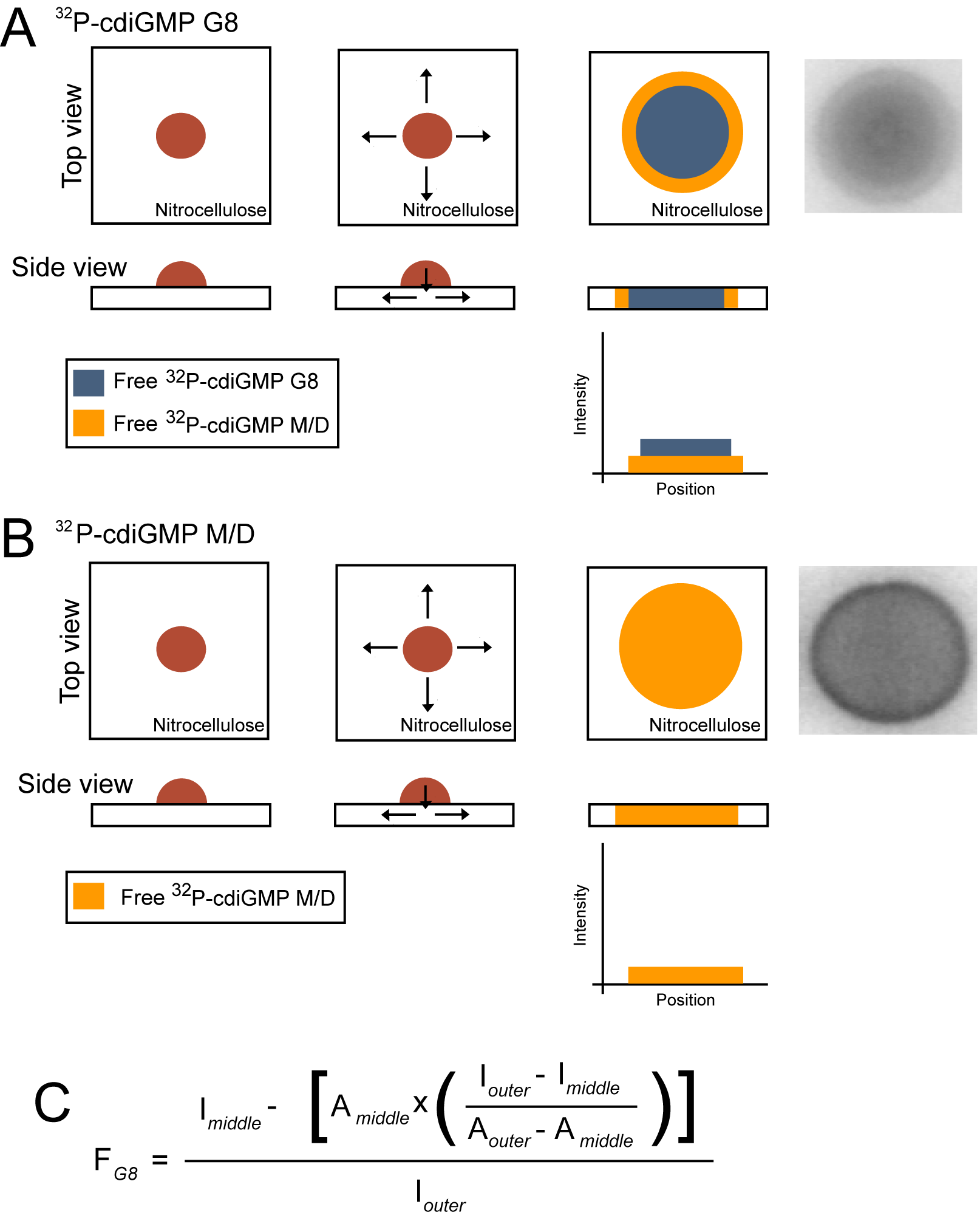

Supplement: Figure S2 — 32P-cdiGMP G8 forms two concentric circles when spotted on nitrocellulose. (A) Schematic representing 32P-cdiGMP G8 spotting on DRaCALA. A 2.5 µL spot of radiolabel without protein is spotted and spreads out radially by capillary action leaving two circles. The 32P-cdiGMP G8 is present in the inner circle while the 32P-cdiGMP M/D is present in both. Box 4, top view shows imaged nitrocellulose after spotting. (B) Diagram representing diffuse radial capillary action of 32P-cdiGMP M/D upon spotting on nitrocellulose. One homogenous circle is observed. Box 4, top view shows an image of 32P-cdiGMP M/D spot (2.5 µL) on nitrocellulose. (C) Equation used to calculate the fraction (F) of 32P-cdiGMP G8 in the mixture. (TIF) [file pone.0053689.s002.tif]

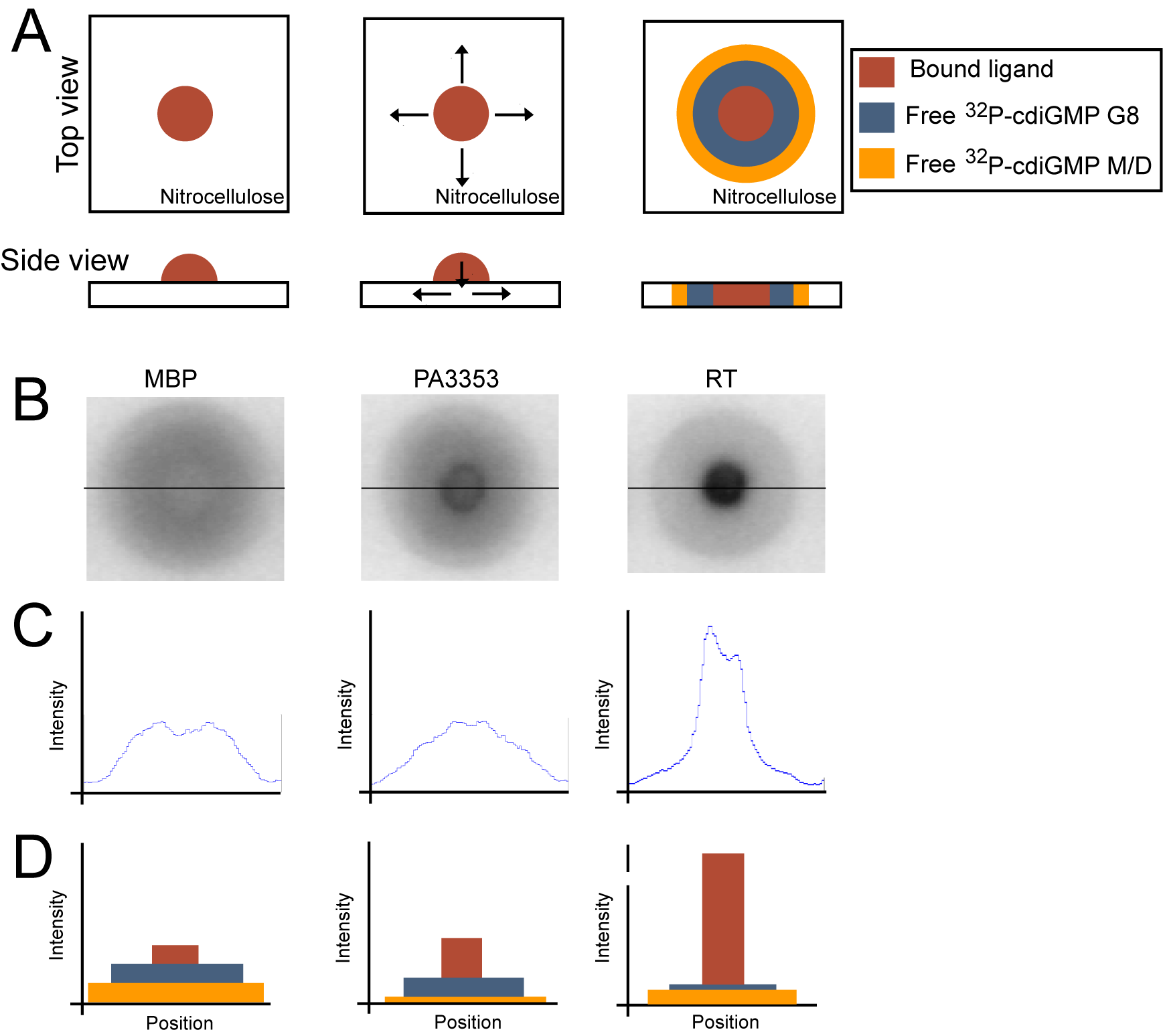

Supplement: Figure S3 — Maltose binding protein (MBP) can be used as a standard to fraction 32P-cdiGMP G8 bound by other proteins. (A) Schematic representing appearance of DRaCALA spot upon addition of protein to the reaction mixture. Inner circle contains sequestered ligand. (B) Imaged DRaCALA spots (2.5 µL) of reactions that included MBP, PA3353, and RT. The line appearing through the image is used for the intensity vs. position plot in (C). (C) Intensity vs. position plots across the black lines in Fig. S2B for binding reactions that included MBP, PA3353, and RT. (D) Cartoon representing idealized intensity vs. position diagrams MBP, PA3353, and RT binding reactions. (TIF) [file pone.0053689.s003.tif]

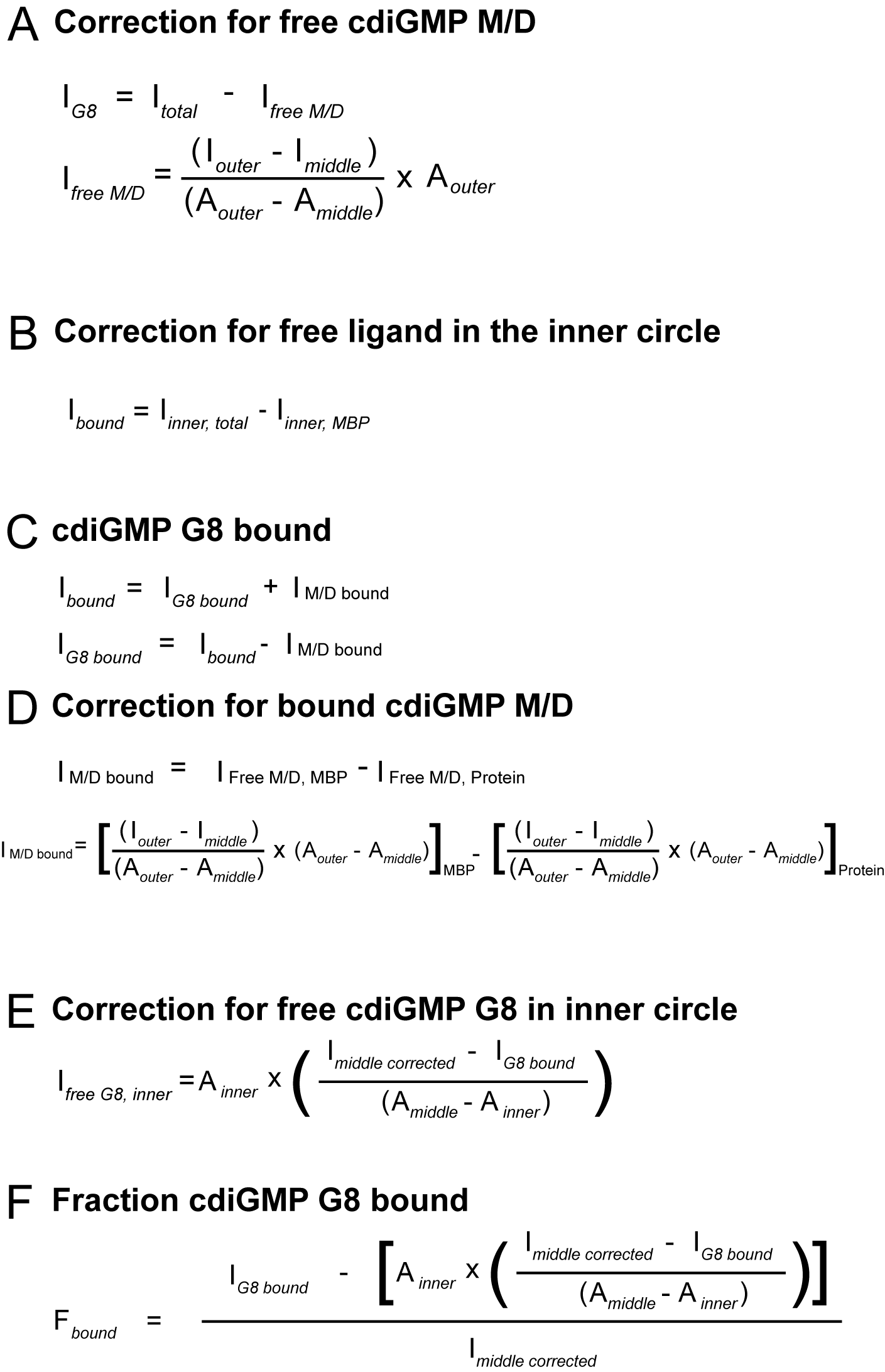

Supplement: Figure S4 — Equations used to calculate fraction 32P-cdiGMP G8 bound by cdiGMP M/D binding proteins. (A) Equations used to subtract free 32P-cdiGMP M/D from the intensity of the entire spot. (B) Correction used to eliminate intensity of MBP inner circle when calculating fraction bound. (C) The total bound intensity is comprised of the intensity of 32P-cdiGMP M/D bound and the intensity of the 32P-cdiGMP G8 bound. (D) The intensity of the 32P-cdiGMP M/D bound can be calculated by subtracting the total unbound 32P-cdiGMP M/D in a sample from the total 32P-cdiGMP M/D in the MBP sample. (E) Equation used to correct for free 32P-cdiGMP G8 that is present in the inner circle. (F) Calculation used to determine the fraction 32P-cdiGMP G8 bound. (TIF) [file pone.0053689.s004.tif]
