# Supplementary material for: Detection of Cyclic Diguanylate G-Octaplex Assembly and Interaction with Proteins
Source: PLoS One. 2013 Jan 7;8(1):e53689. doi: 10.1371/journal.pone.0053689 (PMC3538687; doi:10.1371/journal.pone.0053689)
Supplement: Text S1. — (DOCX) [file pone.0053689.s005.docx]

**Supplemental material**

Stability of cdiGMP M/D and G8 assessed by TLC

CdiGMP (500 µM) was mixed with 4 nM ^32^P-cdiGMP and potassium and either heated as described above and cooled (labeled cdiGMP G8) or not heated (labeled cdiGMP M/D). Beginning when the cdiGMP G8 sample was fully cooled (time = 0), 0.6 µL were removed from the sample and spotted on TLC plates. Remaining sample was incubated at room temperature for the times listed. Samples were then separated as described above and analyzed by phosphorimager (Fig. S1). Fraction cdiGMP G8 and cdiGMP M/D were calculated by dividing the intensity of that spot with the total intensity of the cdiGMP M/D and cdiGMP G8 spots.

Fraction bound calculations for cdiGMP G8 by DRaCALA

When the ^32^P-cdiGMP G8 mixture was spotted on nitrocellulose in absence of protein, two concentric circles containing radiation were observed (Fig. S2A). In contrast, the ^32^P-cdiGMP M/D migrated outward radially into only one observable circle (Fig. S2B). In order to calculate the fraction of ^32^P-cdiGMP G8 bound by protein using DRaCALA, we needed to determine the total intensity of ^32^P-cdiGMP G8 in the mixture [54]. On TLC, the ^32^P-cdiGMP G8 had less mobility while the ^32^P-cdiGMP M/D migrated farther in the hydrophilic buffer used (Fig. 2A). Because molecules are immobilized on nitrocellulose based on hydrophobic interactions, we hypothesized that ^32^P-cdiGMP M/D migrated farther and was present in the outer ring, while both M/D and G8 were present in the middle ring. The presence of two distinct mobility species by DRaCALA complicates the fraction bound calculation. In order to correct for the cdiGMP M/D present, a subtraction must be made from the intensity of the middle circle to remove the signal from cdiGMP M/D (Fig. S2C). This allowed us to quantify the fraction of the slower migrating species to be ~ 75%. Since that value was the same as the fraction ^32^P-cdiGMP G8 as determined by TLC for this specific sample, we concluded that the ^32^P-cdiGMP G8 was the less mobile species on nitrocellulose. Interestingly, when this ligand mixture was spotted with RT, which we have established as a cdiGMP G8 binding protein, the middle circle was no longer present suggesting that the ligand present in the middle circle only was being bound by RT (Fig. S3B, RT). The outer circle remained unaffected by RT which we have shown does not bind cdiGMP M/D. This provides further evidence that the ^32^P-cdiGMP M/D has greater mobility on nitrocellulose.

Having determined that the ^32^P-cdiGMP G8 was present in the middle circle, we were interested in using this to quantify the fraction ^32^P-cdiGMP G8 bound to proteins. The total intensity of the cdiGMP G8 form can be calculated by subtracting the total free cdiGMP M/D from the total intensity of the entire spot (Fig. S4A). The total intensity of ^32^P-cdiGMP M/D is obtained by determining the intensity of the free ^32^P-cdiGMP M/D per unit area in the outer ring and multiplying it by the area of the entire spot (Fig. S4A). Correcting for free ^32^P-cdiGMP M/D leaves only the intensities of bound radiolabel and free ^32^P-cdiGMP G8. The intensity of the "inner circle," where the bound radiolabel is, can be due to sequestration of either cdiGMP G8 or cdiGMP M/D. To distinguish between these two possibilities, we determined the intensity at each position across a single DRaCALA spot for maltose binding protein (MBP) which does not bind either species (Fig. S3C, MBP). The profile of PA3353 which binds cdiGMP M/D was similar to MBP, but the intensity of the outer ring was reduced while the intensity of the inner spot was increased (Fig. S3C, PA3353). In contrast, the profile of RT was distinct from MBP and revealed the intensity of the middle ring was concentrated into the inner circle (Fig. S3C, RT). The relative distribution of the cdiGMP M/D and G8 intensities for each protein is shown in a schematic in Fig. S3D.

For non-binding proteins such as MBP, there was still radiolabel signal within the inner circle. To determine the sequestration of radiolabel ligand due to each binding protein, we subtracted the intensity of the inner circle in the MBP reaction from the inner circle intensity of the other spots (Fig. S4B, first equation). The total intensity of the inner circle can be due to sequestration of either G8 or M/D forms. To simplify this calculation, we assume that the M/D form and the G8 forms are stable and not interchanging during the time of the experiments. This is likely true as cdiGMP G8 are remarkably stable (Fig. 2B) [25]. To determine the amount of M/D that is sequestered into the inner circle, our assumption requires that the M/D is depleted from the outer circle. This difference in M/D intensity can be calculated relative to the M/D present in the MBP spots (Fig. S4D). The ^32^P-cdiGMP G8 bound in the inner circle is equal to the total intensity of the inner circle minus the intensity of the ^32^P-cdiGMP M/D bound (Fig. S4C). Finally, to obtain the fraction ^32^P-cdiGMP G8 bound, a correction must be made for free ^32^P-cdiGMP G8 present in the inner circle (Fig. S4E). This value is then subtracted from the total corrected inner circle intensity and divided by the corrected middle circle intensity to obtain the fraction ^32^P-cdiGMP G8 bound (Fig. S4F).
